# Locally advanced colon cancer with cutaneous invasion: case report

**DOI:** 10.1186/s13104-017-2440-0

**Published:** 2017-03-01

**Authors:** Nádia Tenreiro, Cátia Ferreira, Silvia Silva, Rita Marques, Artur Ribeiro, Paulo Jorge Sousa, Fernando Próspero Luís

**Affiliations:** Department of General Surgery, Centro Hospitalar Trás-os-Montes e Alto Douro, Avenida da Noruega, Lordelo, 5000-508 Vila Real, Portugal

**Keywords:** Colon cancer, Locally invasive, Cutaneous invasion, Case report

## Abstract

**Background:**

Locally advanced colon cancer with direct abdominal wall and skin invasion is an extremely rare finding with most data being derived from case reports, historical autopsy-based or single-center retrospective studies. We present a unique case of a colon cancer with direct cutaneous invasion and colocutaneous fistulization.

**Case presentation:**

Eighty-six year old Caucasian female with multiple comorbidities, referred to Surgical Consultation due to ulcerated skin lesion in the abdomen. She had a long-standing large umbilical hernia but with no previous episodes of incarceration or occlusive symptoms. She denied any digestive or constitutional symptoms. Physical examination showed a large non-reducible umbilical hernia, with an associated painless firm mass within the hernia sac and cutaneous ulcerated growth. Colonoscopy revealed transverse colon cancer (endoscopic biopsy of the tumor and skin punch biopsy confirmed adenocarcinoma of the colon). Computed tomography showed a tumoral mass within the umbilical hernia, with cutaneous infiltration and enlarged regional lymph nodes. Rapid local progression led to colocutaneous fistula with total fecal diversion. We performed an extended right hemicolectomy with *en bloc* excision of the hernia sac and infiltrating cutaneous mass.

**Conclusions:**

In the current era of widespread use of screening colonoscopies, initial diagnosis of locally advanced colon cancer is decreasing. However, this unique case presented an opportunity to recall the advantages of multivisceral resections.

## Background

Malignant cutaneous infiltration has been reported to occur in 0.7–9% of all patients with visceral neoplasms [1–4]. Skin metastasis from colorectal carcinoma has a reported prevalence of 5.8% and is a sign for advanced disease [5]. Cutaneous involvement by direct extension is even rarer. Most data on this subject is derived from historical autopsy-based or single-center retrospective studies. We present a case of a colon cancer with direct cutaneous invasion and colocutaneous fistulization.

## Case presentation

Our patient was an 86-year old Caucasian female with multiple comorbidities. Past medical history included arterial hypertension, heart failure (New York Heart Association class III, stage C), obesity, hypercholesterolemia and hyperuricemia. There was no relevant family history.

She was referred to Surgical Consultation due to a peri-umbilical cutaneous ulceration.

She had a long-standing large umbilical hernia but with no previous episodes of incarceration or occlusive symptoms. She noticed a skin lesion adjacent to the umbilicus in the past 2 months with rapid growth and ulceration. She denied any digestive or constitutional symptoms.

**Fig. 1:**
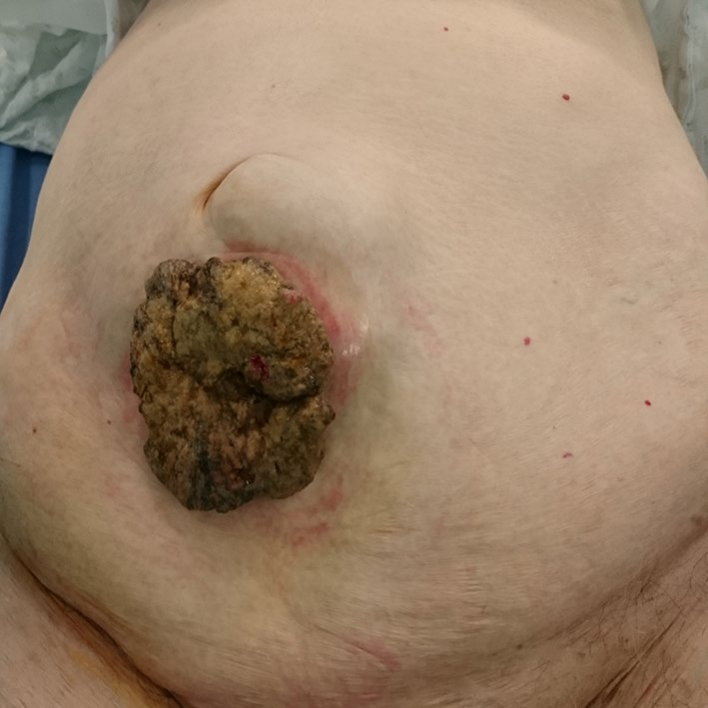
Cutaneous lesion

Physical examination revealed an exophytic cutaneous tumor with approximately 8 cm in diameter (Fig. 1). There was also a large non-reducible umbilical hernia, containing bowel loops and an associated palpable mass within the hernia sac apparently continuous with the cutaneous growth. This hernial mass was painless, firm and adherent to superficial structures and to the abdominal wall, with no pulsatility or fluctuation. There were no other palpable intra-abdominal masses, organomegaly or adenopathy. Examination was otherwise normal.

**Fig. 2:**
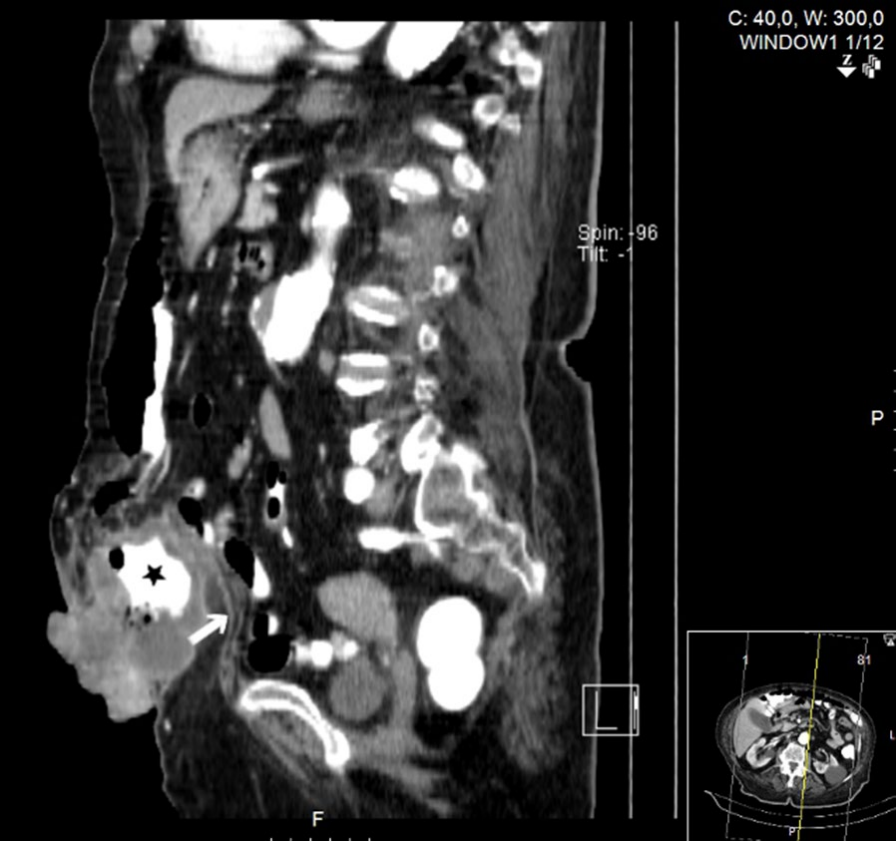
Computed tomography: note the presence of the transverse colon (*star*) within the hernia sac, outside the muscular layer of the abdominal wall (*white arrow*)

Further investigation with colonoscopy revealed transverse colon cancer; endoscopic biopsy of the tumor and skin punch biopsy confirmed adenocarcinoma of the colon. Thoracic and abdomino-pelvic computed tomography showed a tumoral mass within the umbilical hernia, with cutaneous infiltration (Fig. 2) and enlarged regional lymph nodes, but without distant metastasis—cT4bN0M0.

Initially considered for neoadjuvant therapy, rapid local disease progression led to colocutaneous fistulization with total fecal diversion (approximately 1 month), resulting in weight loss and significant quality of life impairment mainly due to colostomy appliance difficulties. The multiple therapeutic options were thoroughly discussed with the patient and family and surgery was decided.

**Fig. 3:**
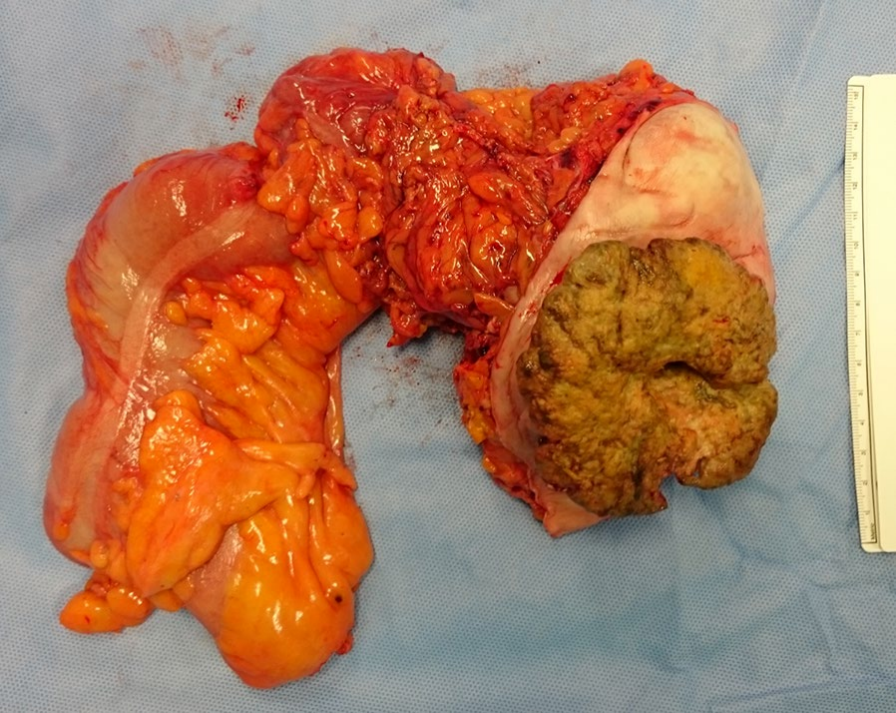
*En bloc* surgical specimen containing colon, abdominal wall and skin

We performed an extended right hemicolectomy with *en bloc* excision of the hernia sac and infiltrating cutaneous mass (Fig. 3). There were no apparent macroscopic intra-abdominal secondary lesions, namely liver or peritoneal metastasis. Considering local conditions and the patients’ co-morbidities we opted for a primary ileocolic side-to-side anastomosis. The remaining rectus sheath was closed primarily without tension, and local advancement skin flaps were necessary for cutaneous closure. Histology revealed moderately differentiated adenocarcinoma of the colon with direct invasion of the abdominal wall and skin. Lymphovascular invasion was present and four lymph node metastasis were identified (out of 12)—stage IIIC (pT4bN2aMx).

Unfortunately, patient status deteriorated after the 4th post-operative day (POD), with cardiovascular shock and progressive multiple organ failure which led to death on the 7th POD. Although no specific imaging evaluation was performed to exclude the possibility of anastomotic dehiscence, clinical and analytical parameters did not favor this diagnosis.

This case report was reported according to CARE guidelines [6].

## Discussion and conclusions

In the ‘90s, a 7316-patient retrospective study showed cutaneous involvement in 2.3% of patients with colorectal cancer, all with metastatic nodules [1]. In a more recent study, 5.8% of patients with colon cancer had skin metastasis with 18.2% present at the time of diagnosis. However, direct skin invasion is rare with no defined prevalence or other epidemiologic data and even case reports are scarce [7, 8].

Approximately 10% of patients with colon cancer have invasion of contiguous organs at diagnosis. Surgical excision remains the mainstay of treatment, although clinical T4b lesions can be considered for neoadjuvant therapy [9]. Multivisceral resection is associated with improved overall survival, being negative margins (R0) the most important prognostic factor [10–13]. Mortality rate seems to be comparable or even lower than single-organ resections [14]. Even so, this approach appears to be underutilized, apparently related to several patient-related factors (sex, age, localization of tumor) and even regional variation [15]. Our patient proved to be a challenge not only because of her multiple comorbidities and advanced age but also due to this bizarre presentation. Ultimately, we decided on a more aggressive approach owing to poor quality of life after the development of the colo-cutaneous fistula. Abdominal wall reconstruction with heterologous material was not necessary.

Screening colonoscopies and overall colon cancer awareness has led to earlier diagnosis and locally advanced cancer is becoming less common. In our case, the unique combination of a transverse colon adenocarcinoma contained in a hernia sac facilitated the direct spread of the tumor. This is an extremely rare finding that reminds us that locally advanced neoplastic disease still presents a challenge and there is still opportunity for improvement.

## Abbreviation

POD: post operative day
